# TB index case tracing in the Roma community in the Czech Republic

**DOI:** 10.1017/S0950268824000384

**Published:** 2024-03-11

**Authors:** Karolína Doležalová, Beatrix Mališková, Michaela Honegerová, Michaela Hromádková, Jiří Wallenfels

**Affiliations:** 1 Clinic of Paediatrics, First Faculty of Medicine, Charles University in Prague and Thomayer University Hospital, Prague, Czech Republic; 2 Pneumology Department, Hospital Mladá Boleslav, Mlada Boleslav, Czech Republic; 3 Regional Public Health Institute of Central Bohemia, Central Bohemia, Czech Republic; 4 National Reference Laboratory for Mycobacteria, National Institute of Public Health, Praha, Czech Republic; 5 National Tuberculosis Surveillance Unit, University Hospital Bulovka, Prague, Czech Republic

**Keywords:** BCG vaccination, contact tracing, public health, Roma community, TB preventive therapy, TB diagnostics, tuberculosis

## Abstract

Tuberculosis (TB) contact tracing and TB preventive treatment are key tools in preventing the transmission of TB with the aim of eliminating the disease. Our study seeks to demonstrate how the infection spread from an individual patient to the entire community and how proactive contact tracing facilitated prompt diagnosis and treatment. Our work was conducted as a retrospective analysis of the spread of TB infection within the Roma community in the Czech Republic, following the case of an index patient who succumbed to pulmonary TB. Several levels of care and preventive and treatment measures are outlined. Confirming the identity of the *Mycobacterium tuberculosis* strain was achieved using molecular methods. Among the 39 individuals examined, TB disease was detected in eight patients and TB infection was detected in six patients. The investigation of contacts within this group yielded positive results in 36% of cases, necessitating treatment. The study’s findings provide evidence that actively tracing individuals at risk can lead to early detection of cases, prompt treatment, and prevention of further disease transmission. The study also indicates that the highest risk of infection occurs within the sick person’s household and that young children under the age of 5 are most susceptible to falling ill.

## Introduction

Tuberculosis (TB) remains a public health priority and is now the leading adult infectious cause of death worldwide. The End TB Strategy calls for bold action to eliminate TB as a public health issue by 2035. Central to the End TB Strategy is the recognition that integrated patient-centred care is required to treat all cases of TB, along with systematic screening of contacts and other high-risk groups to provide effective preventive interventions [1].

Tracing of index TB cases has been advocated for many years as a key component of TB control. It facilitates the early detection of symptomatic and infectious cases and allows people with TB infection to receive preventive treatment [2]. In the Czech Republic, reporting a TB case is a legal obligation of the physician who made the diagnosis/initiated antituberculous treatment. The physician reports the case to the TB register, where it is processed by the Regional Institute of Public Health. Normally, only pneumologists carry out contact investigations and examinations, and the Public Health Institute is only involved in epidemiological investigations in the case of a large or complicated outbreak investigation. The Public Health Institute in the location where the case originated initiates an investigation, as in this case. It forwards the list of contacts to the regional pulmonary clinic, which initiates a clinical investigation, including a chest X-ray (CXR), tuberculin skin test (TST), and/or interferon-gamma release assay (IGRA) test. Patients with positive results are referred to the hospital. There is a double check in the system, as the laboratory where the *Mycobacterium tuberculosis* (*M. tuberculosis*) was cultured also reports culture-positive results to the TB register. If the culture result has no counterpart in the TB register, the Regional Public Health Institute takes steps to ensure that the appropriate source is diagnosed and treated. The care of paediatric TB patients is centralized at one site, the Thomayer University Hospital. World Health Organization (WHO)-recommended preventive TB therapy in paediatric patients is sometimes initiated by a field pneumologist, but more often by an outpatient pneumologist at the paediatric TB centre. In our study, we report a rapid spread of TB infection from an index case to several people in the Roma community and demonstrate cooperation at all levels.

## Material and methods

A retrospective analysis of TB single index case tracing and a special interest investigation to identify those at risk of contracting TB in several levels of public health care were performed. An identical source of infection using molecular biology methods was confirmed.

### Definition of tuberculous infection and tuberculosis

Tuberculous infection (TBI) was defined as a positive result of TST (more than 10 mm) and/or positive result of IGRA test (QuantiFERON TB Gold Plus) and normal findings on CXR without signs of lymphadenopathy, parenchymal lesions, and pleural or miliary changes. TB was defined as a positive result of TST (more than 10 mm) and/or positive result of IGRA test (QuantiFERON TB Gold Plus) and a pathological finding on CXR suggesting TB. In children with positive results of TST and/or positive result of IGRA test (QuantiFERON TB Gold Plus) without a clear pathology suggestive of TB on the CXR, chest computed tomography (CT) was performed.

### Several levels of index case contact tracing

**Figure 1.**
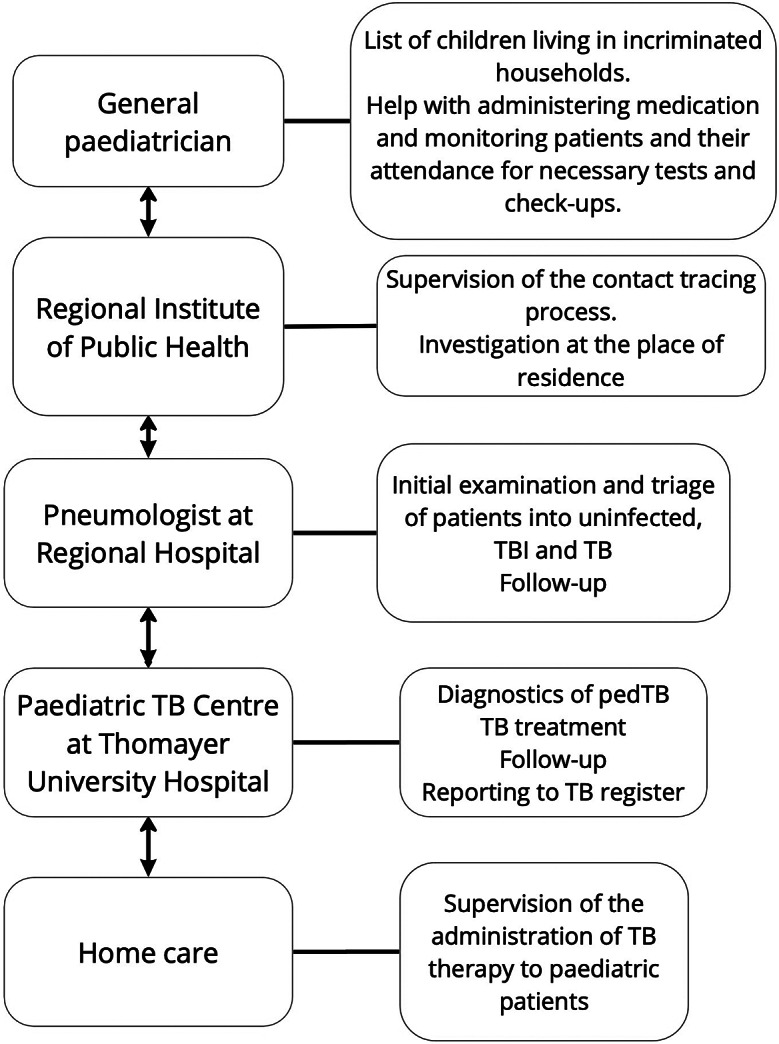
Diagram of the different components of care and their main activities.

As patients from the Roma community denied to have contact with the sick person and to attend the examination, the Regional Public Health Institute of the Central Bohemian Region was involved in the process. In collaboration with the general paediatrician, the regional pneumologist, and the Public Health Institute, a list of people who regularly visited the index case’s household was drawn up. This Roma community has a very social life, so we tried to widen the circle to include everyone who had at least an hour’s contact with the patient. The pneumologist at the regional hospital started an investigation based on this collection of contacts. Each person on the list was examined at least twice, once initially and once 6 months later for adults and 3 months later for children. CXR and TST were performed on all people examined. Most of them additionally underwent an IGRA test. An adult patient with TBI received therapy and ongoing treatment at this outpatient centre. Children diagnosed with TBI or TB were referred to the Paediatric TB Centre at Thomayer University Hospital to commence therapy. All patients with confirmed TB were reported to the TB registry (Figure 1).

### Confirmation of the identity of strain Mycobacterium tuberculosis by molecular methods

Strains from four patients who tested positive and the index case were compared using molecular methods. We utilized the GenoType MTBDRsl ver.2.0 (Bruker) and GenoType MTBDRplus ver.2.0 (HAIN Lifescience) assays. The MTBDRsl assay was used to detect gyrA, gyrB, rrs, and eis genes, along with their primary mutations.

On the other hand, the GenoType MTBDRplus assay was employed to detect rpoB, katG, and inhA genes and their major mutations.

## Results

The index case was that of a 38-year-old man of Roma ethnicity originally from Slovakia, who passed away shortly after being admitted to a district hospital. He had been staying in the Czech Republic for 5 months with his distant family. The patient’s medical records showed weakness, severe weight loss, and cough, with the expectoration of brownish sputum-containing blood. Upon admission, the patient presented with metabolic failure, as evidenced by numerous bilateral chest cavities on X-ray and an abundance of acid-fast rods upon microscopic evaluation of sputum. The sputum test confirmed the presence of *M. tuberculosis*, with negative results for rifampicin resistance genes.

The Regional Public Health Institute was promptly notified and conducted an investigation at the patient’s place of residence, identifying a list of individuals who had been in contact with the deceased party. The investigation revealed that the patient resided in a modest single-storey dwelling shared by three generations of a family with a combined total of six children. On occasions, he stayed overnight with the other family, who had four children, at a separate location (see Table 1). The residents frequently gather in a communal area, and adults smoke. We found that both households were at higher risk of infection because they were in very close contact with the sick person. The other members of the community did not reside in the same abode, and their communication was infrequent. The contact sheet has been transferred to the pneumology outpatient centre, and an investigation into the contacts has been initiated.

**Table 1.** Evaluation of contacts of two households living with the index case

Family member	Age (years)	TST/IGRA	CXR/chest CT	Culture	Dg.
Grandmother	44	Neg.	Neg.		Lost to FU
Grandfather	44	Neg.	Neg.		Lost to FU
Mother	26	Pos.	Neg.	Neg.	TBI
Father	27	Pos.	Neg.	Neg.	TBI
Son 1	7	Pos.,15 mm	Intrathoracic lymphadenopathy	Neg.	TB
Son 2	5	Pos.,18 mm	Intrathoracic lymphadenopathy	Pos.	TB
Son 3	4	Pos.,16 mm	Intrathoracic lymphadenopathy	Neg.	TB
Son 4	2	pos.,18 mm	TB lung infiltration with intrathoracic lymphadenopathy	Pos.	TB
Daughter	9 m	Pos.,10 mm	TB lung infiltration with intrathoracic lymphadenopathy	Pos.	TB
Niece	14	Pos.,12 mm	Neg.	Neg.	TBI
					
Father	22	Neg.	Neg.		Lost to FU
Mother	21	Neg.	Tree in bud	Neg.	Lost to FU
Son 1	2	Pos.,15 mm	TB pleurisy	Neg.	TB
Son 2	4 m	Pos.,12 mm	TB lung infiltration with atelectasis	Pos.	TB
Daughter 1	7	Pos.,16 mm	Neg.	Neg.	TBI
Daughter 2	5	Pos.,18 mm	Intrathoracic lymphadenopathy	Neg.	TB

Abbreviation: FU, follow-up.

A total of 39 individuals underwent testing, comprising 17 adults and 22 minors.

All adults and three adolescents received the Bacillus Calmette–Guérin (BCG) vaccination at birth, and the remaining children were unvaccinated due to the discontinuation of the BCG vaccination programme.

**Figure 2.**
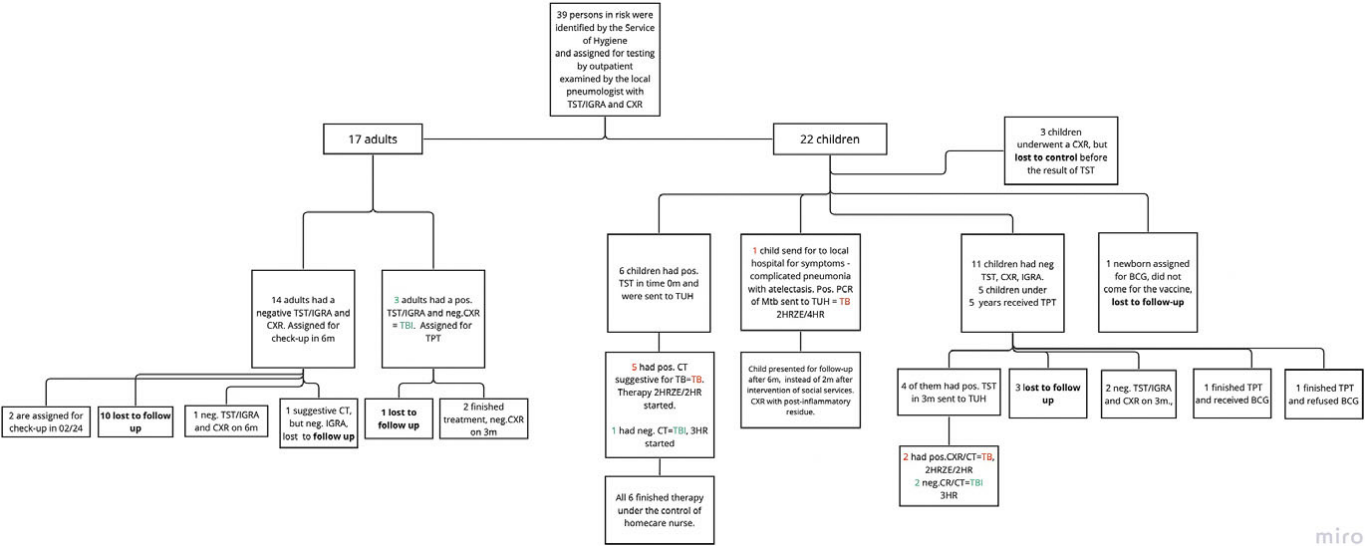
Evaluation of 39 TB index case contacts. Abbreviations: BCG, Bacillus Calmette–Guérin vaccine; CXR, chest X-ray; IGRA, Interferon (IFN)-γ release assay; TB, tuberculosis; TBI, TB infection; TPT, TB preventive therapy; TST, tuberculin skin test; TUH, Thomayer University Hospital.

Fourteen out of 39 subjects tested positive for TST and/or QuntiFERON TB Gold Plus (QTF) and underwent further testing to differentiate TBI from TB. We diagnosed TB in eight people, all children, and TBI in six people (six adults and six children) (see Figure 2).

Of the eight children diagnosed with TB, all shared residency with the index case (refer to Table 1). Four out of eight children diagnosed with TB demonstrated clear pathological changes suggestive of TB in chest CT scans, despite having normal CXRs. These children were subsequently administered the WHO’s recommended short-course TB regimen. Additionally, four out of eight tested positive for *M. tuberculosis* culture.

Prior to contact tracing, a child from a high-risk household was referred to a local paediatric unit for cough and breathlessness. During the examination, CXR revealed extensive atelectasis and right upper lobe opacity. The patient underwent bronchoscopy at the University Hospital, revealing compression in the right upper bronchial area with enlarged lymph nodes. Lavage testing was positive for *M. tuberculosis complex* polymerase chain reaction (PCR). Medical history established family contact with the source case.

Testing of contacts within this group revealed that 36% of all people were positive (14 out of 39), requiring WHO-recommended therapy. Interestingly, 51% of patients were unable to attend follow-up (refer to Figure 2 for further information).

Furthermore, of the eight children diagnosed with TB, only half had a positive culture. All of the cultured strains were compared to the index case strain, with all strains displaying the wild-type binding pattern. As a result, all wild-type probes presented a signal, and therefore, these strains were deemed sensitive to the tested antituberculous therapy. Since all the strains exhibit the same binding pattern, and all the patients reside in the same building, it is probable that they contracted the infection from each other.

## Discussion

The Czech Republic is currently a country with a low TB incidence (3.6 per 100,000 inhabitants in 2022) [3]. Although, in the past the situation in the Czech Republic, respectively, former Czechoslovakia, was the opposite. In 1955, TB prevalence was 1,029/100000 and the mortality rate was 376.1/100000. Factors that have contributed to the radical reduction in overall incidence include improvements in general socioeconomic conditions, the introduction of effective pharmacological treatments, vaccination, isolation of patients, and, last but not least, a well-developed system of patient reporting, index contact tracing, and early initiation of treatment [4]. All these preventive measures have led to a long-term low incidence of TB in the Czech Republic. Since 2010, the country has been able to switch from universal BCG vaccination to a selective vaccination system [5].

At present, TB in the Czech Republic occurs mainly in at-risk populations: homeless people, foreigners from countries with medium-to-high TB incidence (mainly from Ukraine and Vietnam), and the Roma minority. The Roma minority make up about 3% of the Czech population, but they account for 5% of the total number of TB cases in the country as a whole and much more in some districts. The representation of Roma patients among paediatric TB cases is alarming, where they account for almost a third of all paediatric patients [5]. Care for Roma patients is different. Cooperation is usually hampered by a lack of understanding of the severity of the disease, failure to provide preventive therapy, postponement of appointments, and outright exclusion from follow-up [6]. We also share this experience in the context of our study, where all the people studied were of Roma ethnicity and their care had many pitfalls, especially in terms of suboptimal adherence to treatment and a high percentage (51%) of patients lost to follow-up. We are aware that our experience of caring for TB patients in the Roma community is not unique, but rather a common experience. The microepidemy of TB in the Roma community in Prague was described by Křepela in 1990 [7]. In that year, TB spread in the Roma community among six adults, two of whom died at a young age. Fifteen children fell ill. Their treatment was complicated by non-compliance.

The specifics of the spread of TB in the Roma community have also been described by other authors from Eastern Europe. Factors that facilitate the rapid spread of TB in this community include poverty, low standard of living and education, social stigma of TB, smoking, and postponing medical consultations [8–13].

Efforts to improve this situation are already being made by social services, general paediatricians and practitioners, and home care. In our study, we were also able to complete TB treatment in paediatric patients using these components. In the future, the inclusion of Roma outreach workers who would explain the importance of examination, treatment, and medical monitoring in each community and help supervise treatment could help with this issue. A similar network of outreach workers has already been established in neighbouring Slovakia [14].

Our study was designed as a retrospective analysis of index case tracing and led to several conclusions. First, this method of tracing and contact tracing is effective. 36% of those traced were referred for treatment. Early detection of patients and effective treatment prevent further spread of the disease. Second, the largest proportion of patients were young children under the age of 5 living in the same household as the sick person. The youngest children had the most extensive findings on imaging methods. This is consistent with the known fact that the risk of TB transmission is highest in the younger age groups in a shared household and infants are the most at-risk group [15]. Based on the knowledge of the natural development of TB infection, [16] we would expect a predominance of TBI over TB. In this group, there was a significant representation of young children, who are at the highest risk of developing TB. The under-representation of TBI in adult patients may be also partly explained by the fact that many patients were lost to follow-up due to non-compliance and were not diagnosed with TBI. Third, all adults in the household smoked. Smoking is also a risk factor for transmission [17]. Fourth, the children who became ill had not been vaccinated. We know from recent analyses that there may be some protective effect of BCG vaccination, not only against haematogenously transmitted forms of the disease but also to some extent against TBI and TB [18–20]. Fifth, we were surprised by the significant difference in imaging methods. Only 3 out of 10 children had a pathological CXR suggestive of TB. The other children had non-significant CXRs. However, the findings on chest CT showed clear lymphadenopathy or changes in the lung parenchyma. The inadequacy of CXR in depicting lung pathology in children has been demonstrated [21, 22]. In our setting, we currently complement chest CT in all non-specific CXR findings. Sixth, a comparison of the index case strain with other culture-positive patients showed a high probability that it was the same strain. In our setting, where the TB burden is very low, even a larger community can be expected to be infected by a single source of infection. We are aware that our study has several limitations. The present conclusion is based on the experience of only one case. The results presented could not be fully finalized due to the large loss of patients to follow-up. It must also be acknowledged that, despite all efforts, some contacts may have escaped investigation.

## Conclusion

Contact tracing of TB index cases is an effective tool for TB control. In our study, we detected 36% of positive cases requiring treatment. Using molecular biology methods, we were able to compare different strains of *M. tuberculosis* from four culture-positive patients and confirm that they exhibit wild-type binding and are most likely identical pathogens. Tracing of people at risk contributes to early detection of cases, early initiation of treatment, and prevention of further spread of the disease. The Roma ethnic group usually represents an increased risk for the spread of TB. Their compliance is very low, with 51% of people in our study lost to follow-up.

## Data Availability

Data to support the findings of this study are available from the Czech TB Registry for confirmed TB. Access to the TB Registry is not public. If necessary, the TB Surveillance Unit can be contacted (Dr. Wallenfels, co-author of the paper – email jiri.wallenfels@bulovka.cz). The remaining data are available from the authors upon request.
